# Effectiveness of a combined transcranial direct current stimulation and virtual reality-based intervention on upper limb function in chronic individuals post-stroke with persistent severe hemiparesis: a randomized controlled trial

**DOI:** 10.1186/s12984-021-00896-2

**Published:** 2021-07-01

**Authors:** Roberto Llorens, María Antonia Fuentes, Adrián Borrego, Jorge Latorre, Mariano Alcañiz, Carolina Colomer, Enrique Noé

**Affiliations:** 1 Neurorehabilitation and Brain Research Group, Instituto de Investigación e Innovación en Bioingeniería, Universitat Politècnica de València, Camino de Vera s/n, 46011 Valencia, Spain; 2 NEURORHB. Servicio de Neurorrehabilitación de Hospitales Vithas, Fundación Hospitales Vithas, Callosa d’En Sarrià 12, 46007 València, Spain

**Keywords:** Transcranial direct current stimulation, Virtual reality, Eye-tracking, Surface electromyography, Hemiparesis, Stroke

## Abstract

**Background:**

Functional impairments derived from the non-use of severely affected upper limb after stroke have been proposed to be mitigated by action observation and imagination-based techniques, whose effectiveness is enhanced when combined with transcranial direct current stimulation (tDCS). Preliminary studies in mildly impaired individuals in the acute phase post-stroke show intensified effects when action is facilitated by tDCS and mediated by virtual reality (VR) but the effectiveness in cases of severe impairment and chronic stroke is unknown. This study investigated the effectiveness of a combined tDCS and VR-based intervention in the sensorimotor function of chronic individuals post-stroke with persistent severe hemiparesis compared to conventional physical therapy.

**Methods:**

Twenty-nine participants were randomized into an experimental group, who received 30 minutes of the combined tDCS and VR-based therapy and 30 minutes of conventional physical therapy, or a control group, who exclusively received conventional physical therapy focusing on passive and active assistive range of motion exercises. The sensorimotor function of all participants was assessed before and after 25 one-hour sessions, administered three to five times a week, using the upper extremity subscale of the Fugl-Meyer Assessment, the time and ability subscales of the Wolf Motor Function Test, and the Nottingham Sensory Assessment.

**Results:**

A clinically meaningful improvement of the upper limb motor function was consistently revealed in all motor measures after the experimental intervention, but not after conventional physical therapy. Similar limited effects were detected in the sensory function in both groups.

**Conclusion:**

The combined tDCS and VR-based paradigm provided not only greater but also clinically meaningful improvement in the motor function (and similar sensory effects) in comparison to conventional physical therapy.

**Supplementary Information:**

The online version contains supplementary material available at 10.1186/s12984-021-00896-2.

## Background

Functional impairment of the upper limbs is a common sequelae after stroke that affects up to 85% of the survivors [1] and persists, with a certain degree of severity, in 30 to 60% of the cases, six months after the onset, limiting the complete recovery of functional use to only 5 to 20% of them [2, 3]. Given the incidence of upper limb deficits after stroke, and its impact on the participation in activities of daily living [1], social life [4], and quality of life [5], rehabilitation is an imperative goal of physical and occupational therapy.

Although there is no standard intervention for improving upper limb function after stroke [6], functional recovery is believed to occur in response to active exercise and to motor inclusion of the affected limb in task-oriented activities [7, 8]. Consequently, severe impairment of the upper limb function that prevents voluntary movements represents a major challenge to conventional interventions. As proof, less functional recovery is expected from individuals post-stroke who present more impaired motor conditions upon inclusion in rehabilitation programs [9].

Conventional approaches to chronic severe hemiparesis are focused on providing a passive range of motion exercises to preserve the mobility and flexibility of the affected extremity [10] or to compensate for the impaired function by training the less affected limb in unimanual task-oriented exercises [11]. Although passive exercises can produce proprioceptive input to motor pathways [12] and compensation can facilitate some degree of self-sufficiency [11], the absence of self-triggered movements and non-use of the affected limb may lead to a reduced sensorimotor representation in the available neural circuits over time [13] and, consequently, diminish the possibility for clinical improvement [11], an effect that is known as ‘learned non-use’ [14].

Different therapeutic interventions have been proposed to overcome the neural and functional decline caused by this effect, by modulating the excitability of the motor cortex circuitry in the absence of movement [15]. Motor imagery, the mental execution of a movement without any overt movement or muscle activation [16], has been shown to induce a spatial and temporal recruitment of motor cortical areas that mirrors the modulation produced during real motor practice [16–18]. Interestingly, motor imagery is not restricted to individuals with a degree of residual function and, in contrast to passive exercises, still incorporates voluntary drive [16]. Although its application to severely impaired function in individuals with chronic stroke has produced promising improvements [19], stroke can affect the ability to understand and practice different aspects of motor imagery, a technique that is already inherently complex [20]. Mirror therapy, an intervention based on staring at the reflected movements of the non-paretic limb on a mirror placed in the person’s midsagittal plane, as if they were produced by the affected side [21], can potentially overcome the difficulty in imagining the movement, while similarly modulating the activity of the primary motor cortex. Different studies have evidenced an increase in M1 excitability or increased ipsilateral activation, although the findings have been somewhat inconsistent [22]. Although mirror therapy has shown effectiveness in improving motor function in individuals with chronic stroke with mild to moderate impairment [23, 24], its effect on severely impaired individuals with stroke has been reported as being limited to a small effect on tactile sensation [25]. The capacity of motor imagery and mirror therapy to modulate brain activity in the ipsilesional hemisphere is supported by the mirror neuron system theory [26] and suggests that such interventions may be functionally akin to preparatory and executive motor processes [27].

Non-invasive brain stimulation, such as transcranial magnetic stimulation and transcranial direct current stimulation (tDCS) have been proved to modulate cortical excitability through the application of a magnetic field or low-intensity electric current to the scalp using a coil or saline-soaked electrodes, respectively. When applied to the primary motor cortex, it may prime neuroplasticity and motor learning effects [28], which have been shown to improve motor function after stroke [29, 30]. While current evidence suggests a similar potential effectiveness of both techniques [31, 32], the overall lower costs, lower safety risks, and potential to be applied concurrently during rehabilitation of tDCS can facilitate its clinical integration [33]. In contrast to the inconsistent results in its earlier stages, tDCS has shown positive results at improving motor function of the paretic upper limb in chronic stroke [34, 35]. Interestingly, the combination of tDCS and mirror therapy has shown additive effects on motor performance [36] and, similarly, its combination with motor imagery has been reported to modulate not only the neural correlates of movement [37–39], but also the motor performance of upper limb tasks [40, 41].

The addition of tDCS to a motor observation and execution task mediated by virtual reality (VR) has been found to augment motor improvement after stroke [42, 43], which could be supported by an increased short-term corticospinal facilitation [44]. The capacity of VR to provide controlled multi-modal stimulation in one or more sensory channels [45] has also motivated its use in motor observation and imagery [46–48]. Its capacity to allow users to perform virtual movements in a non-physical reality without executing the motor action in the real world is especially interesting, allowing the participation of individuals with severe impairments in the upper limb function in self-triggered tasks, thus closing the loop of interaction-stimulation [49]. Additionally, VR-based interventions alone have shown to promote substantial recovery not only in the acute phase but also within the chronic phase post-stroke [50–52].

Our preliminary studies suggests that a paradigm combining tDCS and a VR-based motor observation task triggered by conscious active responses can provide a feasible and well-accepted rehabilitation framework for individuals with chronic stroke and severely affected upper limb function [53, 54]. We hypothesized that this paradigm could also provide sensorimotor benefits to this population, when compared to conventional physical therapy. The objective of this study was, therefore, to determine the effectiveness of the combined tDCS and VR-based intervention in the upper limb motor and sensory function of severely impaired individuals with chronic stroke in comparison to conventional physical therapy.

## Materials and methods

### Participants

Participants were recruited from the long-term care unit of the neurorehabilitation service at Hospital Vithas Valencia al Mar (València, Spain) and the Brain Injury Centre Vithas Vinalopó (Elx, Spain) from June 2015 to September 2016. The inclusion criteria for participation in the study were: (1) time since injury greater than six months; (2) severe paresis of an upper limb, defined by the Brunnstrom Approach [55] as stages I or II and by the upper extremity subscale of the Fugl-Meyer Assessment [56] as scores below 19; (3) absence of changes in upper limb motor function, as described by the above-mentioned scales, in the last two months; (4) capacity to maintain a sitting position for at least 60 minutes; and (5) fairly good cognitive condition, defined by scores above 23 in the Mini-Mental State Examination [57]. Individuals were excluded if they had: (1) pacemakers; (2) brain implants or other implanted metallic objects (valves, coils, etc.); (3) impaired comprehension that would hinder sufficient understanding of the instructions, defined by scores below 45 in the Mississippi Aphasia Screening Test [58]; (4) severe visual impairments; and (5) emotional or behavioral circumstances that would impede adequate collaboration.

A minimum sample size of 26 participants was required to ensure a power of 0.85, assuming an effect size of 0.25 and an error probability of 0.05. Six additional participants were considered so as to prevent a dropout rate of 20%, accounting for a total of 32 participants.

This study was registered at clinicaltrials.gov (NCT03528018; Retrospectively registered on May 17, 2018) and was approved by the Institutional Review Board of Hospital Vithas Valencia al Mar. All subjects who satisfied the inclusion criteria and accepted the terms of participation in the study provided informed written consent before enrollment.

### Instrumentation

The experimental setup consisted of an interactive VR-based system that provided coherent audiovisual and tactile feedback when an intention of action was detected, while administering concurrent tDCS [53, 54]. Intention of action was interpreted from the gaze and residual muscular activity and movements. Consequently, as the motor observation task was self-triggered, this interaction allowed for active participation in the rehabilitation paradigm.

Gaze was estimated using a portable low-cost eye-tracking bar, the EyeX (Tobii Technology AB, Danderyd, Sweden). This device can estimate the spot on a screen where the user is looking, basing on reflections of infrared light recorded in their pupils [59], at a minimum framerate of 30 Hz in an operating range of 50 to 90 cm. Muscular activity and movement were estimated using a low-cost gesture- and motion-control armband, the Myo (Thalmic Labs, Kitchener, ON, Canada). This device includes seven medical-grade stainless steel sensors that surround the arm and provide surface electromyographic activity (sEMG) [60] at 200 Hz, a three-axis gyroscope that provides angular velocity at 50 Hz, and a three-axis accelerometer that provides acceleration also at 50 Hz. The brachioradialis, palmaris longus, and flexors and extensors of the fingers were expected to be the main potential contributors to the sEMG data.

Audiovisual stimulation was provided by the 15.6-inch screen and two integrated speakers of a laptop. Vibrotactile feedback was provided in the palmar side of the metacarpophalangeal joint of the thumb, index, and pinky fingers using three coin vibrators that were embedded in a hand-made Velcro band. Actuators vibrated independently at 200 ± 40 Hz to simulate collisions with virtual elements. tDCS was provided using the StarStim (Neuroelectrics, Barcelona, Spain), an eight-channel wireless hybrid EEG/tDCS headset that includes a neoprene headcap with 39 fixed positions based on the 10–10 system, where electrodes can be inserted. The headset enables the passage of currents of up to 2 mA, with a resolution of 1 μA, transferred via saline-soaked surface sponge electrodes.

**Fig. 1:**
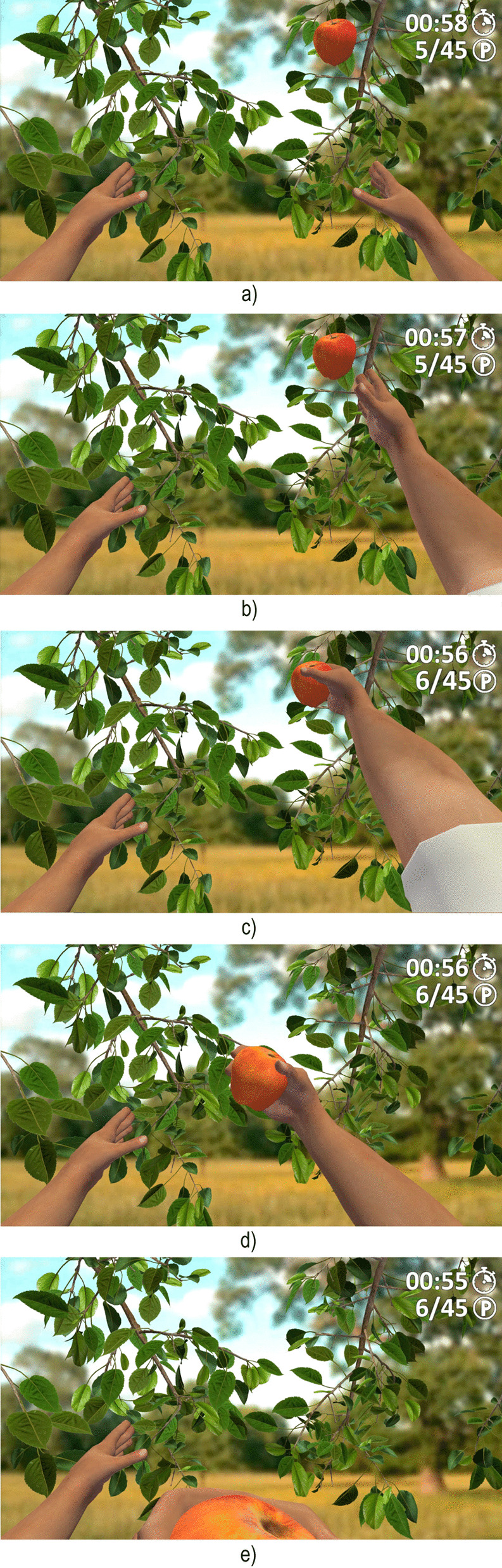
Virtual environment of the self-triggered motor observation task. The self-triggered motor observation task simulated an apple-picking task in an apple orchard (a). If interaction was successful, an animation showed the left or right virtual arm extending towards the apple (b), grasping it (c), bringing it towards the mouth (d), biting it several times (e), and moving the arm to the initial position. The figure represents an animation sequence with the right hand

The VR-based exercise simulated an apple-picking task in an orchard. An egocentric representation of the participants’ arms was located in front of an apple tree, where a serial of apples appeared at four possible fixed locations, on the left and right branches of the tree, disappearing after a few seconds. The object of the exercise was to pick the apples that sequentially grew before they disappeared with the closest virtual hand, either left or right, by performing (or trying to perform) the movement with their real hemiparetic arm. Specifically, participants were required to stare at the apples and try to move their hemiparetic arms as if they were attempting to pick the virtual apples with their real hands. Participants had ten seconds to pick an apple, with the time between apples set at four seconds. An attempt was considered successful if participants stared at the apple for two seconds and if they were able to produce a muscular activity, angular velocity, or acceleration greater than 80% of their maximum values, as registered during the calibration. If so, a winning sound effect was provided and a predefined ten-second animation showed the corresponding virtual arm extending towards the apple, grasping it, bringing it towards the mouth, the mouth biting it several times, and the arm moving back to the initial position (Fig. 1). As the animation was self-triggered, the exercise enabled active participation in the motor observation. Vibrotactile stimulation was also provided, both when the hand grasped the apple and with each bite. On the contrary, an attempt was considered unsuccessful if participants failed at accomplishing the visual, muscular, or motion task. If this case, a losing sound effect, but no vibrotactile stimulation, was provided. If the muscular activity or motion exceeded the required threshold although participants did not stare at the apple, but rather at another point, a six-second animation showed the virtual arm extending towards that point and failing the attempt. Otherwise, the virtual arms remained still. Extrinsic feedback was provided, including the time left, number of repetitions, and record number of repetitions.

### Procedure

Participants were randomly assigned to either a conventional (control) or combined tDCS and VR-based intervention (experimental) group. The randomization schedule was computer-generated, using a basic random number generator in a ratio of 1:1. The allocation sequence was concealed from an independent researcher. A sealed envelope was given to the coordinator of the physical therapy area to identify the group for each participant.

All the participants underwent a total of 25 one-hour sessions, administered three to five times a week. Two-minute breaks were established after each 8-minutes period of therapy. Consequently, the effective time of both the experimental and control intervention was 48 minutes. The participants in the control group received conventional physical therapy. This therapy focused on providing passive range of motion exercises in those segments where no active movement was detected to meticulously reproduce a range of articular movements and muscle and soft tissue elongation. These exercises were manually administered on the affected joints of the hemiparetic side, with the participants in either supine, sitting or standing position, as appropriate. The presence of active movement was investigated after passive exercises. In those segments where residual active movement capability was detected, the participants were encouraged to perform movements with the assistance of the therapists. Passive and active exercises alternated series of activity and rest periods of variable duration. The type of exercises, intensity and duration of the exercises were customized to the particular needs of each participant. Participants in the experimental group combined 30 minutes of the experimental intervention with 30 minutes of the conventional physical therapy program, in that order, which accounted for 24 minutes of effective time spent in each part, 48 minutes in total. The conventional physical therapy administered to this group was also individualized to each participant but its duration was limited to the 30-minutes time frame.

**Fig. 2:**
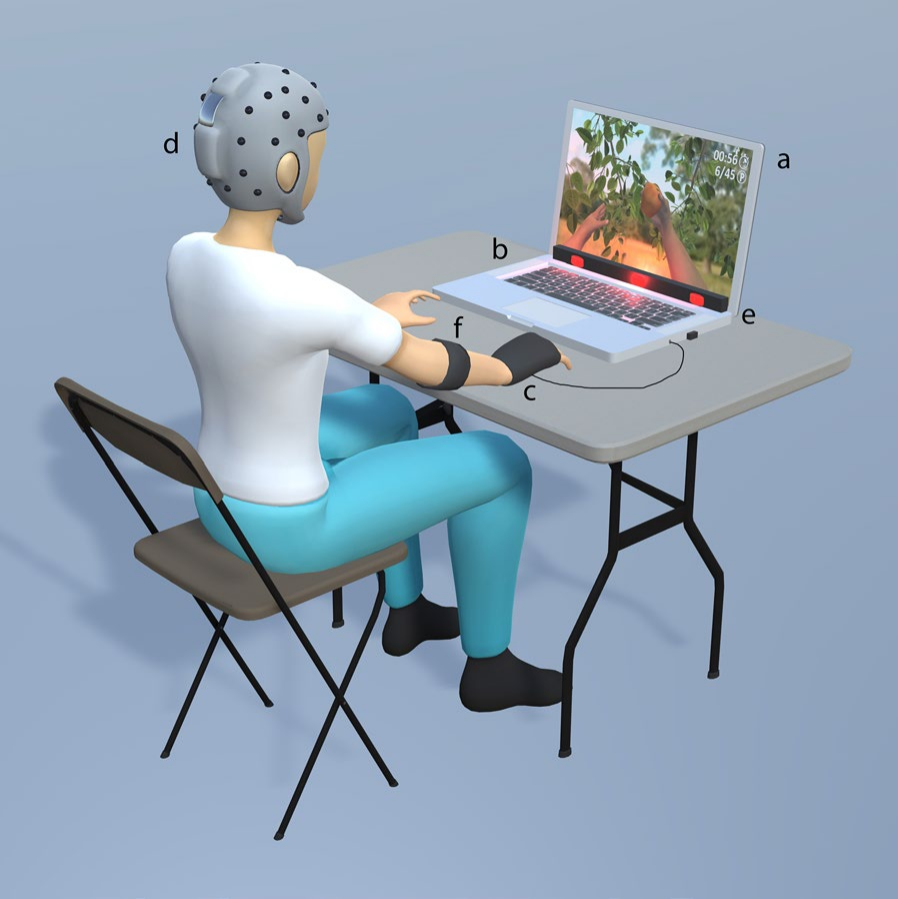
Experimental setup. Participants sat in a chair with their arms on a table, upon which a laptop was located. The intervention provided visual and auditory stimulation through the screen (**a**) and speakers (**b**) of the laptop, respectively, vibrotactile feedback through three independent vibrators (**c**), and transcranial direct current stimulation (**d**). Interaction was allowed through gaze, detected by a portable eye-tracking bar (**e**) and muscular activity and movement, detected by the gyroscopes, accelerometers, and/or surface electromyographic activity of a motion control armband (**f**)

At the beginning of the session, a physical therapist equipped each participant with the EMG bracelet, the vibration band, and the tDCS headband, and the participant sat in a chair with their back leaning against the backrest and their arms on a table located in front of the chair (Fig. 2). The laptop was placed approximately 50 cm from the head and 40 cm below eye-level. The eye-tracker was tilted towards the eyes, and two tDCS electrodes, with a surface of 25 cm^2^ were soaked in saline solution. The anode was placed over the ipsilesional primary motor cortex (M1; C3 or C4 for left or right hemiparesis, respectively) and the cathode was placed in the contralesional supraorbital cortex (Fp2 or Fp1 for left or right hemiparesis, respectively). The impedance was kept below 10 kΩ and the voltage below 26 V. The maximum output intensity was set to 2 mA. The eye-tracking coordinates and the muscular activity and movement thresholds were calibrated to fit the characteristics of each participant [53], and then the session started. tDCS was uninterruptedly provided during the whole session. All the sessions were carried out in a dedicated area of the physical therapy unit. A non-blinded physical therapist supervised all the sessions and prevented extreme compensatory movements, by providing a tactile cue to correct the performance.

The sensorimotor condition of the participants was assessed in a dedicated session before and after the intervention by an external physical therapist who was blinded to the intervention. Outcomes measures were selected following the recommendations of the StrokEDGE Taskforce [61]. The body structure function was evaluated using the upper extremity subscale of the Fugl-Meyer Assessment Scale, which was the primary outcome measure, and the Nottingham Sensory Assessment [62]. The body activities were evaluated using the time and functional ability scores of the Wolf Motor Function Test [63]. The upper extremity subscale of the Fugl-Meyer Assessment Scale has excellent inter and intra-rater [64–68] and test–retest reliability [68–70]. The inter and intra-rater and test–retest reliability of both subscales of the Wolf Motor Function Test are likewise excellent [63, 69, 71, 72]. The reliability of the subscales of the Nottingham Sensory Assessment has been reported from poor to excellent [62, 73].

### Data analysis

The normality of the data distribution was assessed using the Shapiro–Wilk test. All investigated variables were normally distributed. Homogeneity of variance assumptions were assessed with Levene's tests. Demographic and clinical comparisons between groups, and between responders and non-responders, were performed with independent sample t-tests and chi square tests, as appropriate, to investigate comparability at baseline, and indicators of response to the experimental treatment. Mixed factorial analyses of variance (ANOVAs), with time (before and after treatment) as the within-subjects factor and treatment option (control vs. experimental) as the between-subjects factor, were performed for all outcome measures. ANOVA findings that violated the sphericity assumption were accommodated by the Greenhouse–Geisser conservative degrees of freedom adjustment. The main effects of time, treatment option, and the time-treatment option interaction effects were evaluated. Partial eta squared (η^2^_p_) was computed for each ANOVA as a measure of the effect size. Values of effect size may range from 0 to 1, with higher values representing higher proportions of variance explained by the independent variable.

The α level was set at 0.05 for all analyses (two-sided). All treatment effects and partial eta squared analyses were computed by a researcher blind to group allocation using the SPSS Statistics software, version 22 (IBM, Armonk, NY, USA).

## Results

### Participants

**Fig. 3:**
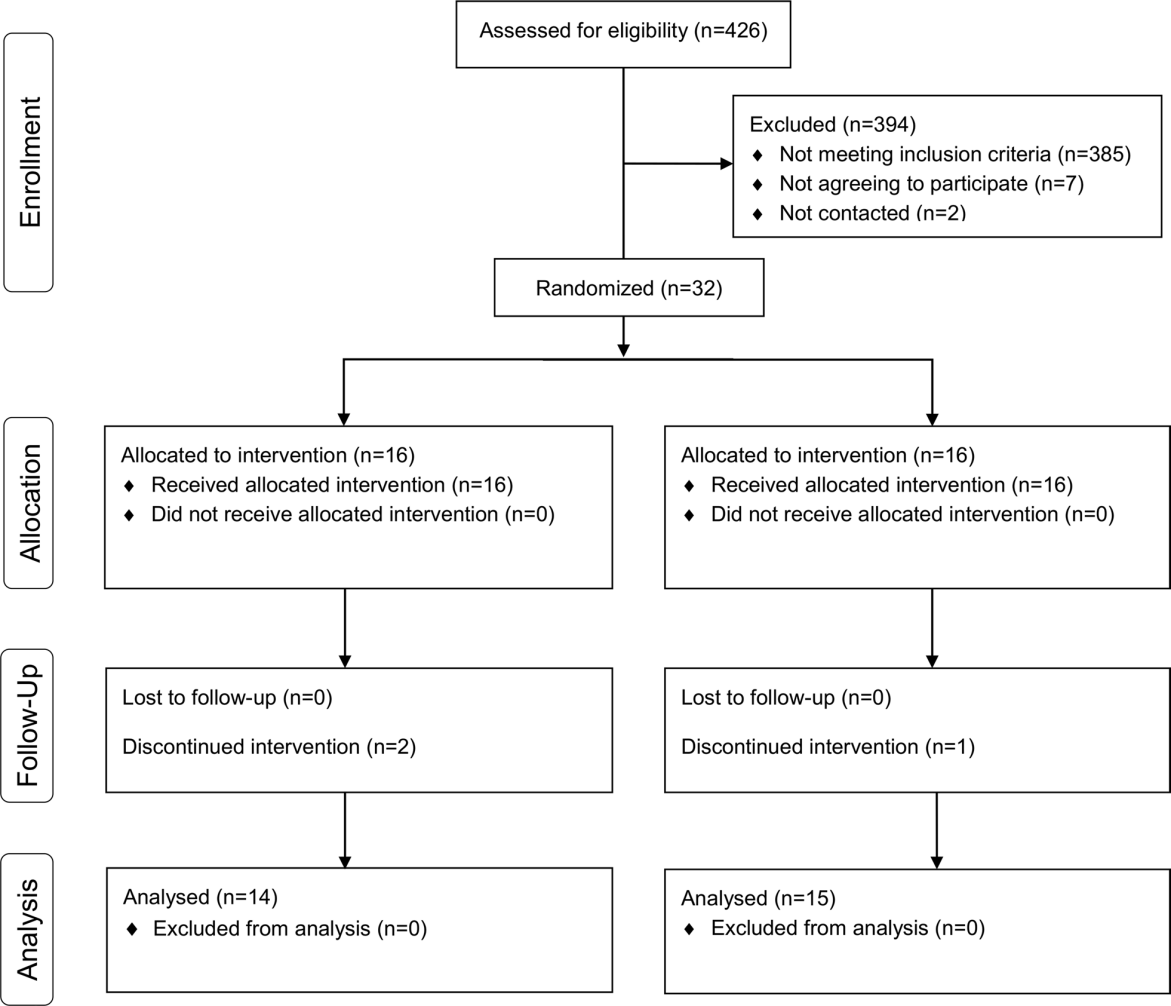
CONSORT flow diagram. Progress through the phases of the parallel randomized trial of both groups

During the recruitment process, a total of 426 outpatients were attending a neurorehabilitation program in one of the facilities (Fig. 3). Of those, 41 (9.6%) individuals met the inclusion criteria for participation in the study. Potential candidates were recorded on a list, and then were randomly contacted and invited to participate in the study until the required sample size was reached. Thirty-nine individuals were finally approached to reach the required sample of 32 participants, as seven of them refused to participate in the study. The 32 individuals who accepted to participate were equally randomized into the control or experimental groups. One participant in the control group had health issues unrelated to the study and could only attend one session during a week, and two participants of the experimental group were discharged and dropped out of the study. Consequently, their data were not included in the analysis. Therefore, the data from 29 participants, 15 in the control group and 14 in the experimental group, were included in this study. The study ended when the last participant completed the intervention.

The final sample consisted of 22 men and seven women, with a mean age of 54.9 ± 9.4 years, and a mean time since onset of 9.0 ± 2.3 months (Table 1). Eight participants presented a hemorrhagic stroke and 21 presented an ischemic stroke. No significant differences were found between groups in terms of demographic (sex and age) or clinical (etiology, hemiparetic side, and time since injury) data at baseline. No significant differences were detected in the clinical scales at baseline. No adverse effects were detected neither in the experimental nor in the control group.

**Table 1:** Characteristics of the participants

	Control group (n = 15)	Experimental group (n = 14)	Significance
Gender (n, %)			NS (p = 0.742)
Male	11 (73.3%)	11 (78.6%)	
Female	4 (26.7%)	3 (21.4%)	
Age (years)	52.3 ± 10.9	57.6 ± 6.9	NS (p = 0.133)
Etiology (n, %)			NS (p = 0.344)
Ischemic stroke	12 (75.0%)	9 (64.3%)	
Hemorrhagic stroke	3 (25.0%)	5 (35.7%)	
Hemiparesis (n, %)			NS (p = 0.599)
Left	11 (36.4%)	9 (55.5%)	
Right	4 (63.6%)	5 (44.5%)	
Time since injury (months)	9.3 ± 2.4	8.7 ± 2.3	NS (p = 0.533)

Gender, etiology, and hemiparesis are expressed as a percentage of the total number of participants. Age and time since injury are expressed in terms of mean and standard deviation. *NS* non-significant

### Motor function

A significant time effect was detected in all measures of upper limb motor function (Table 2). However, improvement provided by the experimental intervention was significantly higher than that provided by the conventional physical therapy. In contrast to participants in the control group, who showed limited improvements, participants who were engaged in the experimental intervention showed consistent improvement, as evidenced by the Fugl-Meyer Assessment Scale (p < 0.001, η^2^_p_ = 0.44), and by the time (p = 0.036, η^2^_p_ = 0.15) and ability subscales (p = 0.043, η^2^_p_ = 0.14) of the Wolf Motor Function Test. Importantly, the improvement experienced by the participants in the experimental group overtook the clinical importance threshold of the three measures, which suggest that the effects of the experimental intervention could be perceived as beneficial and could mandate a change in their clinical management [74]. The improvement in these participants from the Fugl-Meyer Assessment Scale was 5.3 ± 4.1, which exceeded the clinical importance difference established for people with chronic stroke, using this measure [75]. Five of the 9 participants who were classified as being severely impaired before the intervention according to this scale improved their degree of impairment to severe to moderate after the intervention. Improvements in the time and ability subscales of the Wolf Motor Function Test were 7.1 ± 9.3 s and 2.2 ± 2.6, respectively, which also exceeded the clinical importance thresholds established for the chronic stage [76]. However, the effect size of these findings was poor. Improvement in the motor function shown by the participants grouped by etiology of stroke is described in Additional file 1.

**Table 2:** Treatment effects on upper limb sensorimotor function

	Initial assessment	Final assessment	Significance
*Fugl-Meyer assessment scale. Upper extremity subscale*			T**(p < 0.001, η^2^_p_ = 0.49) GxT**(p < 0.001, η^2^_p_ = 0.44)
Control group	9.87 ± 4.82	10.13 ± 4.60	
Experimental group	9.50 ± 5.11	14.79 ± 7.37	
*Wolf motor function test. Performance time (s)*			T**(p = 0.002, η^2^_p_ = 0.30) GxT*(p = 0.036, η^2^_p_ = 0.15)
Control group	100.3 ± 16.8	98.8 ± 18.6	
Experimental group	110.2 ± 13.9	103.1 ± 17.6	
*Wolf motor function test. Functional ability*			T**(p < 0.001, η^2^_p_ = 0.37) GxT*(p = 0.043, η^2^_p_ = 0.14)
Control group	11.60 ± 7.56	12.27 ± 7.65	
Experimental group	8.86 ± 11.77	11.07 ± 13.04	
*Nottingham sensory assessment*			T(p = 0.050, η^2^_p_ = 0.51) GxT(p = 0.598, η^2^_p_ = 0.08)
Control group	31.93 ± 21.44	33.87 ± 21.44	
Experimental group	35.43 ± 24.70	36.57 ± 23.91	
*Nottingham sensory assessment. Tactile subscale*			T*(p = 0.035, η^2^_p_ = 0.58) GxT(p = 0.607, η^2^_p_ = 0.08)
Control group	25.93 ± 18.71	27.53 ± 18.09	
Experimental group	29.71 ± 20.60	30.71 ± 19.37	
*Nottingham sensory assessment. Kinesthetic subscale*			T(p = 0.468, η^2^_p_ = 0.10) GxT(p = 0.955, η^2^_p_ = 0.05)
Control group	6.00 ± 3.50	6.33 ± 4.01	
Experimental group	5.57 ± 4.47	5.86 ± 4.74	
*Nottingham sensory assessment. Stereognosis subscale*			T*(p = 0.041, η^2^_p_ = 0.55) GxT(p = 0.168, η^2^_p_ = 0.28)
Control group	5.27 ± 6.65	5.33 ± 6.66	
Experimental group	4.00 ± 5.16	5.28 ± 6.62	

Clinical data are given in terms of mean and standard deviation.* T* time effect,* GxT* group by time effect. *p < 0.05, **p < 0.01

It is also important to highlight that four individuals out of the 14 participants in the experimental group (28.6%) did not show any motor improvement. This effect was indicated by inexistent (no change) or scant (one-point change) improvements using the Fugl-Meyer Assessment Scale, and was confirmed by an absence of improvements using the subscales of the Wolf Motor Function Test. No clinical or demographic differences were found between these participants and the remaining participants of the experimental group.

### Sensory function

Regarding sensory assessment, neither the total score (p = 0.598, η^2^_p_ = 0.08) nor the tactile (p = 0.607, η^2^_p_ = 0.08), kinesthetic (p = 0.955, η^2^_p_ = 0.05), or stereognosis subscales (p = 0.168, η^2^_p_ = 0.28) of the Nottingham Sensory Assessment showed significant differences between groups (Table 2). No differences between groups emerged when analyzing the items of the tactile subscale (light touch, temperature, pinprick, pressure, tactile localization, bilateral simultaneous touch) separately. A significant time effect was detected in the tactile and stereognosis subscales in both groups, which showed comparable improvements after the intervention. Improvement in the sensory function shown by the participants grouped by etiology of stroke is described in Additional file 1.

## Discussion

This study investigated the effectiveness of a combined tDCS and VR-based intervention on upper limb function in chronic individuals post-stroke with persistent severe hemiparesis in comparison to a conventional physical therapy intervention. Our results provide evidence that participation in self-triggered motor observation tasks, coupled with tDCS, can provide clinically meaningful improvements in the motor function when compared to conventional physical therapy alone, while having similar effects on the sensory function.

Improvement in the motor function detected in the body structure, evidenced by the Fugl-Meyer Assessment Scale, is supported by similar interventions combining tDCS and VR in individuals with acute stroke and mild impairment, with changes of up to 10 points on the same scale reported [77, 78]. Although the amelioration experienced by the participants was less dramatic, the relevance of their progress should be highlighted due to their time since injury and the severity of their impairment, as both attributes have been reported to limit the expected benefits of post-stroke interventions [9, 79]. Improvements detected in the body activities, evidenced by the Wolf Motor Function Test, are consistent with the results from the body structure. The enhancement of both performance time and functional ability are not only supported by previous reports, but also exceeded the reported benefits obtained by mildly impaired individuals in the acute phase post-stroke [78]. However, interpretation of the results in this scale should be done with caution considering the small effect size detected in our study. Although the remarkable progress of the participants in our study could be explained by their greater room for improvement, as they presented severely impaired function, it should be taken into account that the severity of the motor function is also the worst prognostic factor for upper limb functional recovery [9]. The improvement detected in the motor function after the experimental intervention might have a particular clinical relevance for the treatment of individuals with the most chronic and severe hemiparesis, as the detected changes were not only statistically significant, but also were clinically meaningful. Importantly, although statistical significance is required to validate any treatment effect, the clinical relevance of the statistical findings is often arguable and, in some cases, very limited [80]. The overcoming of the minimal clinically important difference of both measures of motor function after the experimental intervention support that the improvements in this ability could be effectively perceived by the participants and mandate a change in their clinical management. The detected changes, translated into the clinic, meant that some individuals who were not able to perform hand, wrist, or multi-joint movements and had no movement from single joint extensor and flexor muscle synergies before the experimental intervention, showed certain capacity, although being very limited, to perform these movements after the intervention. The general improvement in motor function after the combined intervention of tDCS and VR is also supported by previous reports on the efficacy of both techniques, tDCS [81–83] and VR [46, 51] applied individually. Importantly, additional improvements have been reported when they have been combined [77], as in this study, which has been argued to facilitate corticospinal excitability [44]. Although this possibility was not explored in the present study, the anodal stimulation over the ipsilesional motor cortex together with the concurrent involvement in the VR-based task and the subsequent participation in physical therapy could have facilitated the motor improvement detected in our participants. Interestingly, this paradigm has been proved to weaken the excitability of intracortical inhibitory circuits concurrently with practice, a phenomenon referred to as gating, which can increase the excitability of the motor cortex during motor learning [84]. The lack of a sham condition in the administration of the tDCS and the impracticability of providing a feasible placebo of the VR intervention could have influenced the effectiveness of the experimental intervention. However, the provision and strength of the sham condition in tDCS is still a matter of debate [85, 86].

The absence of motor changes evidenced by the four non-responders to the intervention are unlikely to be related to demographic nor clinical factors, as no differences in any variables were found that could explain the ineffectiveness of the intervention. We speculate that the absence of effect in these participants could rather be explained by a possible loss of corticospinal tract integrity. A pathological tract disruption could have affected the anatomic connectivity of white matter pathways [87], consequently affecting their motor function. In line with this, the fiber number ratio has been shown to have significant correlation with motor function, which, interestingly, is stronger in the chronic phase than in earlier phases [88]. Moreover, the integrity of the corticospinal tract is the second major predictor of upper limb recovery [9]. Unfortunately, as a limitation of the study, no structural nor functional data was available to check the integrity of the corticospinal tract of the participants, or to determine the effects of the intervention on cortical excitability or reorganization.

The improvements in tactile sensation and stereognosis, evidenced by the Nottingham Sensory Assessment, in all participants could be due to an effect of the passive range of motion exercises administered in both groups, which was not reflected in the motor function. Importantly, passive exercises have been shown to produce proprioceptive input to motor pathways [12], but neither induce improvement in motor performance nor cortical plasticity, as active movement does [89]. The absence of differences between groups might be explained by a lack of specificity in the intervention in addressing the sensory function. The provision of haptic feedback, in the form of vibration, could have positively contributed to motor learning, as a beneficial effect of adding another sensory modality [90], but may have no effect on the sensory function. Although it has been reported that tactile perception can be augmented by viewing the stimulated parts [91], the focus of the attention of the participants on their motor performance, at the expense of sensory integration, may have prevented greater improvements to be derived from the experimental intervention. Interestingly, attention to touch can recruit somatosensory cortical areas, including the primary somatosensory cortex [92]. However, the effect of mirror therapy in individuals with comparable characteristics has shown very small effects on the sensory function (nor in the motor function) [93].

The clinical relevance of our results should be highlighted, based on the following considerations. First, all participants were chronic. The improvement detected in their motor function several months after stroke, where spontaneous neural recovery is unlikely [94], supports the effectiveness of the intervention. Previous reports on the effectiveness of combining tDCS and VR are focused on the acute phase post-stroke [77, 78] and, consequently, are unable to isolate the source of the improvement. Second, the participants also presented residual severe impairment in their upper limb motor function, which, as mentioned above, represents the worst scenario for functional recovery [9]. Third, the overcoming of the minimal clinically important difference in the improvement experienced by individuals who interacted with the combined tDCS and VR paradigm not only supports the relevance of the gains, but could also mandate a change in their clinical management [74]. Fourth, preliminary studies using a comparable protocol have also shown maintenance of gains in upper limb function one month after the intervention [53, 54], and a good acceptance [53]. And finally, the impressive improvement detected in this study provides support to the absence of a rehabilitation plateau, which has been previously suggested [95, 96], as the participants showed no benefits from physical therapy in the last two months prior to the intervention. The inexistent progress in this period might reflect an adaptation to the therapy, rather than being indicative of a diminished capacity for improvement. Inclusion in the experimental intervention could have helped them to overcome the adaptive state by modifying their regimen aspects using novel and different parameters and modalities [95].

The improvement detected in motor function after a combined tDCS and VR-based intervention, together with the good acceptance of the intervention and the potential to provide long-term benefits [53, 54], could support its use as a feasible therapeutic alternative to the scant existing therapeutic options available to chronic individuals post-stroke with severe impairment in the upper limb function, which have provided only a limited effect on the motor function [93], poor acceptance [97], and high cognitive demands [98].

## Conclusions

This study investigated the effectiveness of a combined tDCS and VR-based intervention on upper limb function in individuals with chronic stroke and persistent severe hemiparesis. Our results provide evidence that a combined intervention, consisting of tDCS and a self-triggered motor observation exercise mediated by VR, produced clinically meaningful improvements in the motor function of those individuals compared to conventional physical therapy alone, while having similar effects on sensory function.

## Supplementary Information

**Additional file 1.** Treatment effects on upper limb sensorimotor function according to etiology of stroke. This additional file describes the improvement evidenced by the participants grouped by etiology after the conventional and experimental intervention.

## Data Availability

The datasets used and/or analysed during the current study are available from the corresponding author on reasonable request.
