# Supplementary material for: Effectiveness of a combined transcranial direct current stimulation and virtual reality-based intervention on upper limb function in chronic individuals post-stroke with persistent severe hemiparesis: a randomized controlled trial
Source: J Neuroeng Rehabil. 2021 Jul 1;18:108. doi: 10.1186/s12984-021-00896-2 (PMC8252292; doi:10.1186/s12984-021-00896-2)
Supplement: Supplementary file 1 — Additional file 1. Treatment effects on upper limb sensorimotor function according to etiology of stroke. This additional file describes the improvement evidenced by the participants grouped by etiology after the conventional and experimental intervention. [file 12984_2021_896_MOESM1_ESM.pdf]

Treatment effects on upper limb sensorimotor function according to etiology of stroke

This Additional file describes the improvement evidenced by the participants grouped by etiology after the conventional and experimental intervention. Data are provided in a dedicated table and are also depicted in figures.

Additional Table 1. Mean improvement in the sensorimotor function detected in the participants grouped by etiology

	Conventional intervention	Experimental intervention
Fugl-Meyer Assessment Scale. Upper Extremity subscale		
Ischemic stroke	0.17±1.02	5.89±4.22
Hemorrhagic stroke	0.67±1.15	4.20±4.09
Wolf Motor Function Test. Performance time (s)		
Ischemic stroke	-28.27±46.88	-116.78±147.25
Hemorrhagic stroke	-0.38±0.66	-86.40±137.83
Wolf Motor Function Test. Functional ability		
Ischemic stroke	0.83±1.03	2.78±2.99
Hemorrhagic stroke	0.00	1.20±1.64
Nottingham Sensory Assessment		
Ischemic stroke	2.25±4.27	1.00±7.87
Hemorrhagic stroke	0.67±1.53	1.00±0.00

Clinical data are given in terms of mean and standard deviation.

Additional figure. Mean improvement in the sensorimotor function detected in the participants grouped by etiology

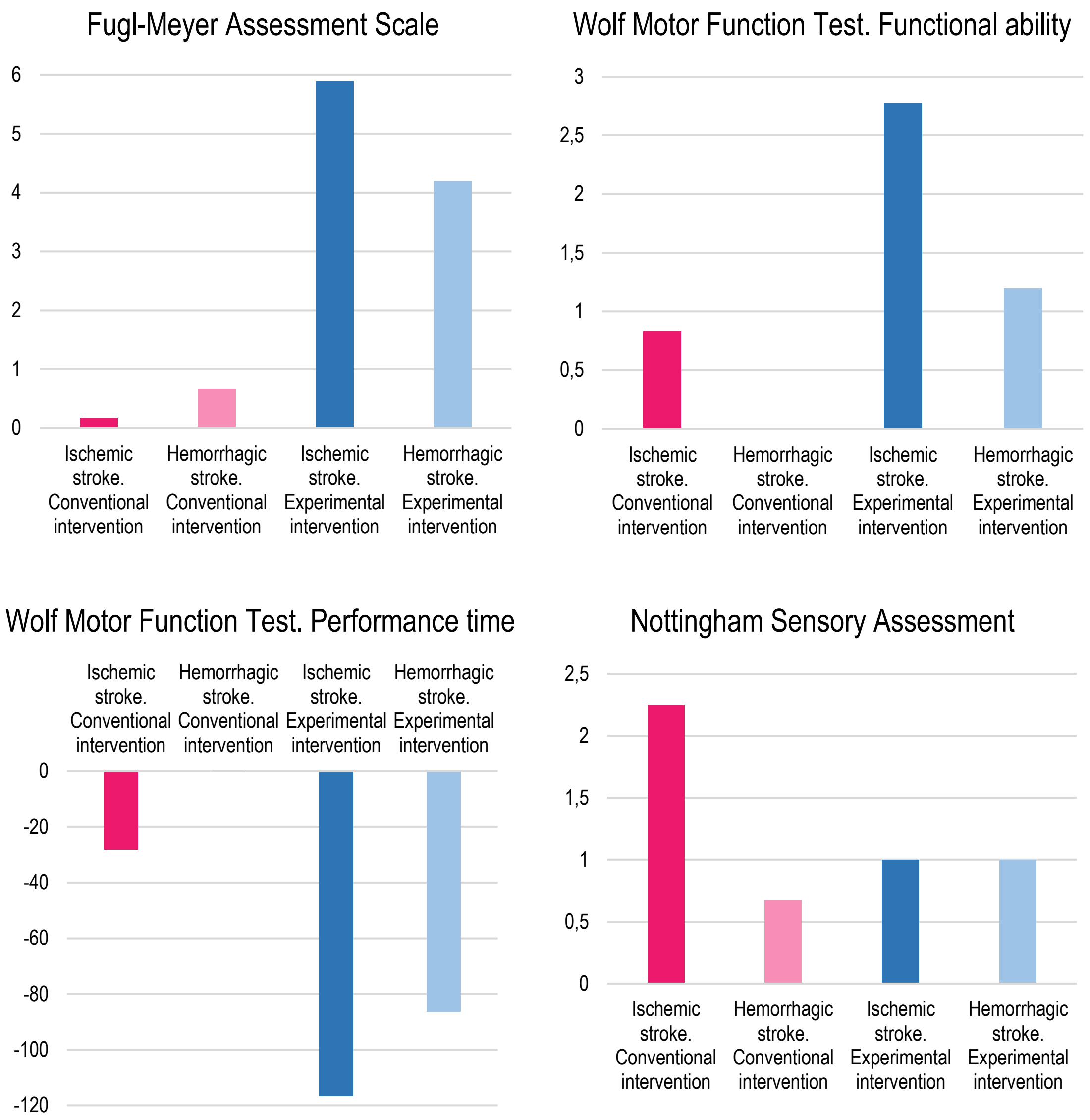

Different shades of pink and blue represent the conventional and experimental intervention, respectively.
